# A New Method for GNSS Multipath Mitigation with an Adaptive Frequency Domain Filter

**DOI:** 10.3390/s18082514

**Published:** 2018-08-01

**Authors:** Siqi Yu, Fei Guo, Xiaohong Zhang, Wanke Liu, Xin Li, Renpan Wu

**Affiliations:** 1 School of Geodesy and Geomatics, Wuhan University, 129 Luoyu Road, Wuhan 430079, China; sqyu@whu.edu.cn (S.Y.); xhzhang@sgg.whu.edu.cn (X.Z.); wkliu@sgg.whu.edu.cn (W.L.); lixinsgg@whu.edu.cn (X.L.); 2011301610244@whu.edu.cn (R.W.); 2 Collaborative Innovation Center for Geospatial Technology, 129 Luoyu Road, Wuhan 430079, China

**Keywords:** GNSS, multipath effect, spectral analysis, short-time Fourier transformation (STFT), adaptive frequency domain filter

## Abstract

Multipath is the dominant error source for most fixed Global Navigation Satellite Systems (GNSS) sites and stations. The presence of multipath, particularly the multipath effect on pseudorange measurement, seriously affects positioning accuracy. Unfortunately, multipath effect reduction is still a challenging issue in high-accuracy GNSS positioning applications due to its special properties. To minimize the impact of the multipath effect, this paper focused on pseudorange multipath mitigation. First, the frequency spectrum of the code-minus-carrier divergence (CMCD) for Global Positioning System (GPS), Global Navigation Satellite System (GLONASS), and BeiDou Navigation Satellite System (BDS) satellites observed in different environments were analyzed, where we found that periodic fluctuations appeared in GPS and GLONASS as well as medium earth orbit (MEO) and inclined geosynchronous satellite orbit (IGSO) of BDS satellites in some situations, which manifested as peaks in the frequency domain. The results showed that the location of the frequency peaks in the frequency domain, width, and basic frequency spectrum intensity were different between different satellites and environments, causing difficulty in reducing the error impact. To eliminate such period fluctuations mainly caused by the multipath effect, a novel method based on a frequency domain filter was proposed in this paper. One of the keys of the proposed method was the use of short-time Fourier transformation (STFT) in the GNSS signal data processing to calculate the accurate local frequency spectrum when code-minus-carrier divergence (CMCD) was assumed to be time varying. Once the frequency spectrum was obtained, a new spectrum peak extraction method was used to locate the peak frequency position. By interpolation and inverse Fourier transformation, the influence of the spectrum peaks could be effectively eliminated, thus improving pseudorange precision. The experimental results showed that the periodic multipath effect could be greatly reduced by the proposed method.

## 1. Introduction

Various kinds of errors affect Global Navigation Satellite Systems (GNSS) positioning, such as ionospheric error, tropospheric error, orbital error, satellite and receiver clock errors, multipath error, and receiver noise. Among these items, multipath has been proven to be a limiting factor, which is difficult to be deal with. Surrounding objects generate many signal replicas that cause a large multipath disturbance in received signals, resulting in large positioning errors [1]. The characteristic related to the environment makes multipath difficult to model and then correct. In most high-precision applications, multipath remains the dominate error source [2]. Therefore, the importance of multipath has led to extensive investigations and implementations of various mitigation methods for over several decades [3,4,5,6,7].

Recently, multipath correction methods have generally been divided into hardware and software categories. Hardware methods include antenna-based methods and receiver hardware modification methods. Antenna-based methods consist of methods utilizing multipath restrained antennas and multiple-antennas systems [8,9]. The chokering antenna is one of the multipath restrained antennas, and has been widely used in multipath reduction [10,11]. Kesler and Haykin proposed a method to estimate the total effect of multipath from all sources on Global Positioning System (GPS) carrier phase data based on multiple antennas [12]. Receiver hardware-based methods concerned more with correlation tracking and multipath estimation delay the lock loop. Fenton proposed one of the first low-cost receivers by employing the narrow correlation tracking technique [13]. In 1992, Dierendonck described the theory and performance of narrow correlator technology [14]. Townsend and Fenton introduced a tracking loop that took full advantage of the narrow correlator spacing design [15]. 

Software methods include similarity-based methods, selection-based methods, and filter-based methods. Similarity-based methods are one kind of classic method in high-precision multipath determination. If a receiver observes a GPS signal for a long time, the multipath signature can be identified by examining a daily repeated pattern in the received signal. This technique is suitable when static GPS receivers are used to observe the same GPS satellite for several days continuously [16,17]. Extensive research has shown that multipath is positively associated with elevation angle and signal-to-noise ratio values [18]. A basic selection-based method to reduce multipath uses satellites with a higher elevation angle or signal-to-noise ratio values [19]. The time domain low-pass filter is able to restrain the high-frequency noise effects on the frequency domain and thus reduce the impact of multipath [20,21,22]. Ge et al. proposed the use of bandpass finite impulse response (FIR) filters to extract and eliminate multipath based on continuous GPS observations [23]. Azarbad and Mosavi proposed a kind of code multipath reducing method based on stationary wavelet transform (SWT) [24]. Mcgraw developed a “Generalized Divergence-Free Smoothing” framework for carrier phase smoothing of GPS pseudorange measurements [25]. Based on code-minus-carrier analysis, Barton and Zhang proposed a kind of frequency domain code multipath mitigation technique for real-time applications [26,27]. Kim et al. proposed the use of a multipath model derived by an estimated ionospheric delay rate to control the Hatch filter parameter in code multipath mitigation [28].

The hardware category method cannot eliminate the multipath error completely. In addition, hardware inaccessibility is the main drawback of hardware-based approaches for most users. For software methods, the selection-based method relies heavily on precision of previously solved solutions and the stability of the environment, and this method may waste useful information in current measurements with multipath. These characteristics lead to the inability of this method to reduce high-dynamic multipath and inefficiency in real-time applications. The challenging issue for the filter-based method is that the filter should be adaptive; since fixed filters cannot cope with the multipath effect due to its time-dependent variation, optimized filter length and step size selection play a critical role in filter design. Another problem that should be considered in the filter-based method is that the GNSS signal and the filter spectrums might lie on the same frequency range, which leads to the removal of the GNSS signal. In this paper, we compared the code-minus-carrier divergence observed by the same type of receivers in different places. We found that waveform fluctuation appeared in the pseudorange measurements when the observed environment was not ideal, which performed as the spectrum peak in low frequency. Analysis results showed that this kind of peak width, peak location, and overall frequency spectrum strength were different among different satellites and environments, and led to difficulty in multipath elimination. One approach to mitigate the threat of this kind of waveform is to use frequency domain filters. Based on the frequency spectrum analysis derived by short-time Fourier transformation (STFT), an adaptive frequency domain filter method was proposed in this paper. The method used STFT to decompose the code-minus-carrier divergence [29,30]. By identifying the location of the frequency spectrum peaks adaptively, the influence of waveform multipath fluctuations can be extracted and then used to correct observations within other frequency features retained maximally. 

This paper contributes to GNSS multipath mitigation with an adaptive frequency domain filter. Section 2 presents the new adaptive frequency domain filter algorithm aimed at periodic fluctuation in detail. Followed by a short statement about the data and processing schemes in Section 3, the experiment results and analysis are shown in Section 4, while Section 5 concludes the paper. 

## 2. Adaptive Frequency Domain Filter Algorithm

Time-domain smoothing and the FIR filter method are traditional methods used for multipath mitigation. The filters used should be adaptive since fixed filters cannot cope with the multipath effect due to its time-dependent variation. Optimized filter length and step size selection are critical for filter design. In real applications, due to the influence of different circumstances, the characteristics of periodic fluctuations cannot be known exactly in advance. To be specific, the period, amplitude, and initial phase of fluctuation varies differently in different satellites and different situations. Therefore, it is difficulty to establish a common multipath model and correct it. A traditional time-averaging filter and low pass FIR filter cannot solve this problem well. The adaptive filter uses a transfer function controlled by variable parameters and a means to adjust those parameters according to an optimization algorithm [31,32,33,34]. Variable parameters such as a smoothing window are often used in the time domain filter [35,36], while filter coefficients are often used to control in the frequency domain filter such as the least mean squares filter (LMS), etc. [37]. The adaptive method is flexible and has self-adaptability to fit many complex conditions.

By dividing a longer time signal into short segments of equal length and then computing the Fourier transform separately on each short segment, STFT can reflect the frequency spectrum characteristics accurately when the signal is time-varying. In this paper, aiming at the time-varying characteristic of period fluctuation, STFT was used to determine the accurate frequency spectrum. Then, based on the frequency spectrum results derived by CMCD, a new adaptive frequency domain filter was presented which can effectively cope with the situation without the period, the amplitude, or the initial phase of fluctuation known in advance. Unlike traditional adaptive methods, this method is intuitive, so is able to find out the abnormal frequency peak location adaptively from the frequency spectrum, and then eliminate the influence of the period fluctuation. Basically, the proposed method focuses on extracting accurate frequency spectrum peaks adaptively and mitigating the multipath effectively by applying an effective algorithm structure.

### 2.1. Procedure of Adaptive Frequency Domain Filter

The procedure of the proposed method mainly includes the following seven steps:

1. CMCD extraction: Using the code and carrier phase measurement as input, the CMC series can be extracted. Then, by differencing the CMC in adjacent epochs, the CMCD series can be obtained.
2. STFT: With the derived CMCD, the frequency spectrum is calculated based on STFT.
3. Spectrum peaks location: Using the real-time STFT frequency spectrum, the left rising slope threshold and right falling slope threshold can be obtained adaptively. Then, the positions of the spectrum peaks can be extracted.
4. Spectrum interpolation: After the peak location is extracted, the peak is cut off by employing interpolation methods, which include the linear interpolation method, cubic spline interpolation, subsection Hermite interpolation method, and cubic interpolation method, etc. In this paper, the cubic interpolation method was used.
5. Fourier inversion: Fourier inversion based on inverse fast Fourier transform (IFFT) is employed for the smoothed spectrum data, then the smoothed frequency-domain data is converted into time-domain data. After Fourier inversion, the corrected CMCD series can be obtained.
6. Initial phase correction: Using the corrected CMCD series and former CMCD series as input, differential values of the CMCD and corrected CMCD can be obtained, and the initial phase value can be corrected afterwards.
7. Pseudorange restoration: Using the differential series of CMCD and corrected CMCD, the corrected CMC series can be obtained by integration. Then, the waveform fluctuations in the pseudorange can be corrected by using the corrected CMC series and initial phase.

**Figure 1:**
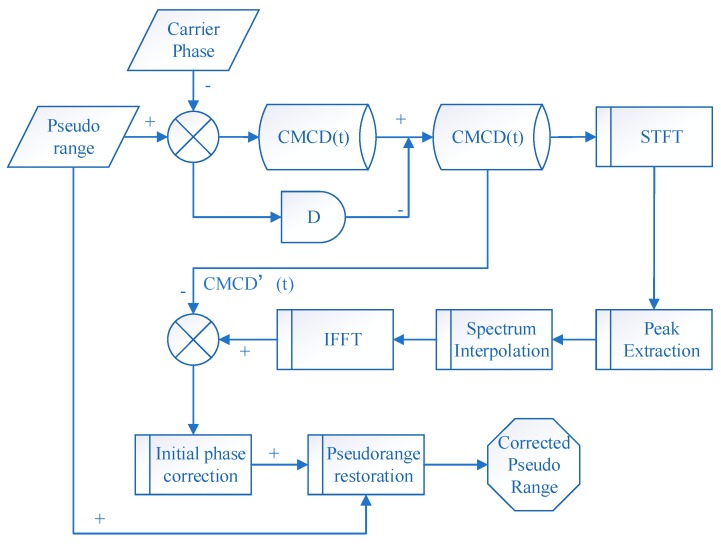
Flow chart of the correction model.

The flow chart of the CMCD correction model is shown in Figure 1. 

### 2.2. Code-Minus-Carrier Divergence

By differencing the code and carrier phase measurement, common errors of code and carrier phase can be eliminated and only those errors that are unique to the code or carrier phase remain. Code and carrier phase measurements can be modeled as:

(1) $$P_{i}=\rho_{i}+I_{i}+T_{i}+c(dt_{i}-dt^{i})+c(d_{i}+d^{i})+X_{i}+e_{i}$$

(2) $$L_{i}=\rho_{i}-I_{i}+T_{i}+c(dt_{i}-dt^{i})+w_{i}+\lambda (f_{i}+f^{i})+\lambda N_{i}+X_{i}+\varepsilon_{i}$$

where $P_{i}$ is the measured code measurement and $L_{i}$ is the measured carrier phase measurement. $\rho_{i}$ is the true distance between the satellite and receiver. $I_{i}$ and $T_{i}$ are the ionospheric delay and tropospheric delay, respectively. $dt^{i}$ is the satellite clock bias. $dt_{i}$ is the receiver clock bias. $d^{i}$ is the satellite pseudo hardware delay. $d_{i}$ is the receiver pseudo hardware delay. $w_{i}$ is the phase wind-up error of the receiver. $f^{i}$ is the satellite carrier hardware delay. $f_{i}$ is the receiver carrier hardware delay. X refers to the errors that can be corrected by models such as phase center offset (PCO), phase center variation (PCV), relativity, etc. $e_{i}$ is the non-parametric error of code which contains the pseudo multipath and random error. $\varepsilon_{i}$ is the non-parametric error of the carrier phase which contains the carrier phase multipath and random error.

By merging the same type of items, the code and carrier phase measurements can be reconstructed as:

(3) $$P_{i}={\dot{\rho }}_{i}+I_{i}+c(d_{i}+d^{i})+e_{i}$$

(4) $$L_{i}={\dot{\rho }}_{i}-I_{i}+w_{i}+\lambda (f_{i}-f^{i})+\lambda N_{i}+\varepsilon_{i}$$

where ${\dot{\rho }}_{i}=\rho_{i}+T_{i}+c(dt_{i}-dt^{i})+X_{i}$, denoting the common part of $P_{i}$ and $L_{i}$.

From Equations (3) and (4), the code-minus-carrier (CMC) observation reads as:

(5) $$Z_{i}(k)=P_{i}-L_{i}=2I_{i}+c(d_{i}+d^{i})-w_{i}-\lambda (f_{i}-f^{i})+\lambda N_{i}+e_{i}-\varepsilon_{i}$$

In addition, Equation (5) can be further simplified as:

(6) $$Z_{i}(k)=P_{i}-L_{i}=2I_{i}+Y+\lambda N_{i}+{\varepsilon_{i}}^{\prime }$$

where $Y=c(d_{i}+d^{i})-w_{i}-\lambda (f_{i}-f^{i})$, ${\varepsilon_{i}}^{\prime }=e_{i}-\varepsilon_{i}$; Y denotes the sum of the fixed bias, which is considered to be stable in a period of time; ${\varepsilon_{i}}^{\prime }$ represents the sum of random errors.

By differencing the CMC in adjacent epochs, we obtain:

(7) $$\begin{array}{ll} CMCD_{i}(k) & =Z_{i}(k)-Z_{i}(k-1)\\ & =2{\dot{I}}_{i}+{\varepsilon_{i}}^{\prime }(k)-{\varepsilon_{i}}^{\prime }(k-1) \end{array}$$

As shown in Equation (7), the common items are effectively eliminated, leaving only the influence of ionospheric gradient, multipath, and random errors in CMCD [38].

### 2.3. Spectrum Peak Extracting Method

**Figure 2:**
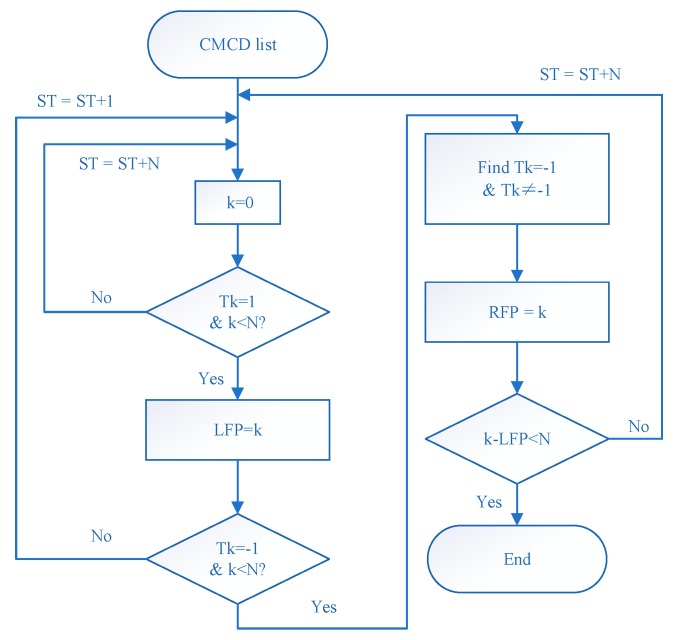
Flowchart of the peak extracting method.

Extracting the spectrum peaks accurately is critical for this algorithm. The formation of a peak includes the left inflection point and right inflection point. The two points appear in pairs and cannot exist independently. The distance between two points is the peak width. Then, the spectrum peak extracting method flowchart can be shown, as in Figure 2.

1. Turn the frequency spectrum calculated by STFT into a continuous spectrum. Then, the spectrum slope $T_{k}$ can be calculated. In this paper, glide window technology was used to turn the former spectrum into the continuous spectrum. Then, the significant random fluctuation could be decreased, thus the major variation tendency was kept in a continuous spectrum. The window length of glide window technology used in this experiment was 100.
2. Calculate the left rising slope threshold and the right falling slope threshold. These details are presented in Section 2.4.
3. Turn the continuous spectrum sequence into 0–1 series. If $T_{k}$ is greater than zero, then compare $T_{k}$ with $T_{L}$. If $T_{k}$ is greater than $T_{L}$, then mark the output value as 1, otherwise mark it as zero. If $T_{k}$ is less than zero, then compare $T_{k}$ with $T_{R}$; if $T_{k}$ is greater than $T_{R}$, then mark it as zero, otherwise mark it as –1.
  (8) $$\left\{ \begin{array}{l} T_{k}>0,\;\left\{ \begin{array}{l} T_{k}>T_{L},\;TL(k)=1\\ T_{k}<T_{L},\;TL(k)=0 \end{array}\right.\\ T_{k}<0,\;\left\{ \begin{array}{l} T_{k}>T_{R},\;TL(k)=0\\ T_{k}<T_{R},\;TL(k)=-1 \end{array}\right. \end{array}\right.$$
4. Find out the first $T_{k}=1$ as the left peak point, and start counting the number of epochs. When the epoch number is less than the preset peak width N, step into the next time interval if there is no $T_{k}=-1$. When the $T_{k}=-1$ point is found and continuously equal to −1, set the last point equal to −1 as the right peak point. Then, the position of the frequency spectrum peak can be found. It is worth mentioning that the peak searching region should not be greater than N. If epoch counting reaches N without any point equal to −1 or the range between the left peak point and the right peak point is greater than N, stop searching and turn to the next region.

### 2.4. Adaptive Threshold Setting Combined Strategy

To guarantee the threshold setting combined strategy feasibility in complex situations, the threshold setting strategy includes two methods. 

#### 2.4.1. Adaptive Threshold Method

From the analysis above, we can see that the spectrum increased linearly in the low frequency part. Normally, the mean value and variance trend upwards uniformly. This property is the applicable basis of this kind of adaptive method. Set $N_{w}$ as the frequency spectrum smoothing coefficient, and [a,b] as the frequency spectrum reference range, where $a\geq N_{w}$, $b<N_{\max}-N_{w}$, $N_{\max}$ is the max frequency spectrum width. In each subinterval, calculate the subinterval mean value list μ(k) and subinterval variance list σ(k) using the spectrum amplified in each subinterval, which can be shown as follows, where k is the frequency sampling point sequence.

(9) $$\left\{ \begin{array}{l} \mu (k)=\frac{1}{N_{w}}\cdot \sum_{i=k-N_{w}/2}^{k+N_{w}/2-1}h(i)\\ \sigma (k)=\frac{1}{N_{w}-1}\cdot \sum_{i=k-N_{w}/2}^{k+N_{w}/2-1}h{\left(i\right)}^{2} \end{array}\right.$$

Use the corresponding frequency list f(k) and μ(k), wherein first-order linear fitting is used to calculate the mean value slope $k_{\mu }$ and mean value bias $b_{\mu }$, which can be shown as:

(10) $$\mu (k)=k_{\mu }\cdot f(k)+b_{\mu }$$

Similarly, use f(k) and σ(k), wherein the fitting equation of f(k) and σ(k) can be shown as Equation (11), in which $k_{\sigma }$ and $b_{\sigma }$ indicate the variance slope and variance bias.

(11) $$\sigma (k)=k_{\sigma }\cdot f(k)+b_{\sigma }$$

Let τ denote the amplification factor. Then, the left threshold list and right threshold list can be shown as:

(12) $$\left\{ \begin{array}{l} T_{L}(k)=\mu (k)+\tau \cdot \sigma (k)\\ T_{R}(k)=\mu (k)-\tau \cdot \sigma (k) \end{array}\right.$$

This kind of adaptive threshold method performs well when the abnormal peak frequency location is not too low and the abnormal peak amplitude is not too high. The low frequency threshold is relatively sensitive. When the abnormal frequency is located in an extremely low frequency, and if the fitting range includes the abnormal frequency, the fitting parameter deviation is great and the fitting results are not ideal in this situation. When the abnormal peak amplitude is too high, the fitting result may be strongly influenced by the abnormal points, and this may result in the wrong $k_{\mu }$ and $b_{\mu }$, thus deriving the wrong threshold. The threshold method described in Section 2.4.2 is employed to solve the insufficiency that occurs when the peak frequency location is too low or the amplitude is too high.

#### 2.4.2. Preset Threshold Method

In this part, based on empirical data, first-order linear fitting was also used to reflect the relationship between the frequency and continuous spectrum. τ times the linear fitting value are used as the left rising slope threshold, while the right falling slope threshold can be calculated similarly. Then, the preset threshold can be shown as:

(13) $$\left\{ \begin{array}{l} T_{L}(f)=\mu_{L,0}+\tau \cdot k_{0}\cdot f\\ T_{R}(f)=\mu_{R,0}-\tau \cdot k_{0}\cdot f \end{array}\right.$$

where $\mu_{L,0}$ and $\mu_{R,0}$ are the preset up and down bias parameters, and $k_{0}$ is the preset slope parameter. f is the frequency sampling point.

These kinds of preset parameters tend to be slightly conservative in order to meet the requirements of different situations. If the parameters are too conservative, the small peaks are hard to detect, which means that this kind of method may not have an obvious effect in situations of small periodic fluctuation. If the thresholds are too strict, the normal situation may also be treated as abnormal peaks, and the useful information may result from being filtered in this situation.

This kind of method is effective when an abnormal peak appears in an extremely low frequency or the abnormal peak amplitude is too high. The reason for this is that the variation range of the low frequency is small, and the presetting parameter would not be influenced by the adaptive fitting deviation. However, when the variation tendency differs from the actual data, the peak detecting performance degrades. Overall, there is great complementarity in the two methods. By combining the results of the two threshold setting methods, complex situations can be improved.

#### 2.4.3. Parameter Influence in Adaptive Threshold Setting Combined Strategy

**Figure 3:**
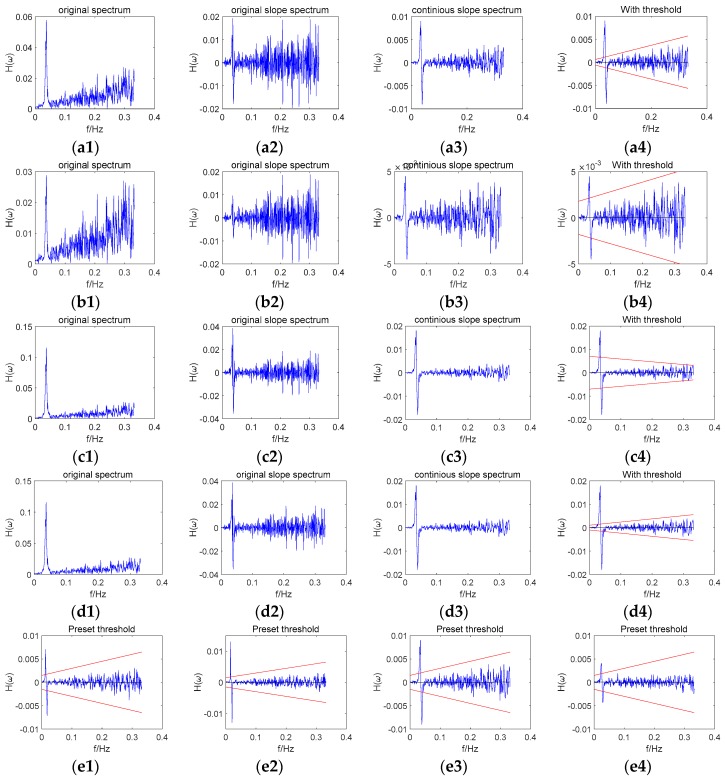
Parameter influence in the adaptive threshold setting combined strategy. (**a1**) original spectrum, [50, 340], 0.06, adaptive, (**a2**) original slope spectrum, [50, 340], 0.06, adaptive, (**a3**) continuous slope spectrum, [50, 340], 0.06,adaptive, (**a4**) comparison with threshold, [50, 340], 0.06, adaptive, (**b1**) original spectrum, [1, 340], 0.03,adaptive, (**b2**) original slope spectrum, [1, 340], 0.03, adaptive, (**b3**) continuous slope spectrum, [1, 340], 0.03, adaptive, (**b4**) comparison with threshold, [1, 340], 0.03, adaptive (**c1**) original spectrum, [1, 340], 0.12, adaptive (**c2**) original slope spectrum, [1, 340], 0.12, adaptive (**c3**) continuous slope spectrum, [1, 340], 0.12, adaptive (**c4**) comparison with threshold, [1, 340], 0.12, adaptive (**d1**) original spectrum, [50, 340], 0.12, adaptive, (**d2**) original slope spectrum, [50, 340], 0.12, adaptive (**d3**) continuous slope spectrum, [50, 340], 0.12, adaptive, (**d4**) comparison with threshold, [50, 340], 0.12, adaptive (**e1**) comparison with threshold, C06, (**e2**) comparison with threshold, C07, (**e3**) comparison with threshold, R04, (**e4**) comparison with threshold, G02.

In order to analyze the influence of parameters in the adaptive threshold method, several examples are given in Figure 3. In Figure 3, (a1), (b1), (c1), and (d1) are the original spectrums in different situations; (a2), (b2), (c2), and (d2) are the original slope spectrums calculated by (a1), (b1), (c1), and (d1); (a3), (b3), (c3), and (d3) are the continuous slope spectrum figures, while (a4), (b4), (c4), and (d4) are the comparisons of the continuous slope spectrums and the thresholds in different situations. The upper red line represents the left rising threshold, while the bottom red line stands for the right falling threshold. (e1), (e2), (e3), and (e4) are the example of a preset method in different situations. In (a1), (a2), (a3), and (a4), the original spectrum of a data series was presented in (a1). In (a2), the original slope spectrum was calculated based on the original spectrum. In (a3), glide window technology was used to calculate the continuous spectrum. In (a4), the adaptive threshold was presented in red lines within the slope spectrum. In (a1), (a2), (a3), and (a4), the adaptive threshold method was used and the frequency spectrum reference range was set as [50, 340] (in point); the peak amplitude is about 0.06; we could find that the threshold method works well in this experiment. In (b1), (b2), (b3), and (b4), the same frequency spectrum was used and the peak amplitude was reduced by half; the adaptive threshold method was used and the frequency spectrum reference range was set as [1, 340] (in point). We found that the threshold tends to be conservative due to the influence of the abnormal peak covered in the frequency spectrum reference range. In (c1), (c2), (c3), and (c4), the same frequency spectrum as (a1) was used and the peak amplitude was doubled; the adaptive threshold method was used and the frequency spectrum reference range was set as [1, 340] (in point). We found that the fitting parameters were wrong and the fitting slopes were smaller than zero. In (d1), (d2), (d3), and (d4), the same frequency spectrum as (c) was used, and the frequency spectrum reference range was set as [50, 340] (in point). In this case, we found that this adaptive method worked well. In (e1), (e2), (e3), and (e4), examples of the preset method in different situations were presented. From the analysis above, we concluded that the correct setting of the frequency spectrum reference range has a great influence on the adaptive threshold method performance. When the abnormal peak was covered in the frequency spectrum reference range, the frequency spectrum amplitude significantly influenced the fitting results. The preset threshold method only gave a conservative threshold, when the adaptive method would not work.

### 2.5. Correction Models

The periodic fluctuations in the time domain are shown as frequency peaks in the frequency domain, which can be expressed as a trigonometric function. Considering the influence caused by a single frequency peak, the pseudorange can be expressed as:

(14) $$P_{i}={\dot{\rho }}_{i}+A\cos(\omega t+\psi )+\varepsilon_{i}$$

where A is the amplitude of the spectrum peak, ω is the frequency of the spectrum peak, and ψ is the phase angle in the present epoch.

Considering that this kind of frequency spectrum peak usually appears in the pseudorange instead of the carrier phase measurement, CMC can be expressed as follows:

(15) $$\begin{array}{ll} Z_{i}(k) & =P_{i}-L_{i}\\ & =2I_{i}+Y+\lambda N_{i}+A\cos(\omega t+\psi )+{\varepsilon_{i}}^{\prime } \end{array}$$

Thus, *CMCD* can be set as:

(16) $$\begin{array}{ll} CMCD_{i}(k) & =Z_{i}(k)-Z_{i}(k-1)\\ & =2{\dot{I}}_{i}-A\omega \sin(\omega t+\psi )+{\varepsilon_{i}}^{\prime }(k)-{\varepsilon_{i}}^{\prime }(k-1) \end{array}$$

After the frequency domain filtering, the influence of the frequency spectrum peaks is eliminated. Then, the corrected *CMCD* can be expressed as:

(17) $$CCMCD_{i}(k)=2{\dot{I}}_{i}+{\varepsilon_{i}}^{\prime }(k)-{\varepsilon_{i}}^{\prime }(k-1)$$

By subtracting Equation (16) from Equation (17), the influence of the frequency peak is obtained as Equation (18):

(18) $$\begin{array}{ll} dU(k) & =CCMCD_{i}(k)-CMCD_{i}(k)\\ & =A\omega \sin(\omega t+\psi ) \end{array}$$

Then, Equation (18) is integrated. Notice that the initial phase should be taken into account in the pseudorange correction. The initial phase A and amplified ω can be calculated by −U(k). After correcting the influence of the initial phase and integration dU(k), we obtain:

(19) U(k)=−Acos(ωt+ψ)

By adding Equation (19) to Equation (14), we obtain the corrected pseudorange as:

(20) $$CP_{i}=P_{i}+U(k)={\dot{\rho }}_{i}+\varepsilon_{i}$$

Therefore, the influence of the frequency spectrum peak can be effectively eliminated.

## 3. Datasets

**Figure 4:**
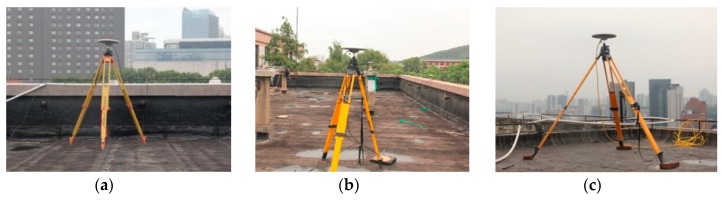
Observation environment and GNSS antennas used in the experiments. (**a**) Station A, (**b**) Station B, (**c**) Station C.

**Figure 5:**
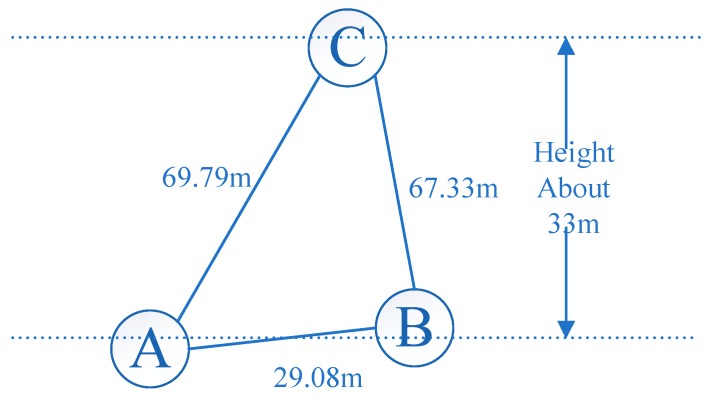
Diagram of the relative positions of the three stations.

Dual-frequency GNSS (Global Positioning System (GPS), Global Navigation Satellite System (GLONASS), BeiDou Navigation Satellite System (BDS)) data were recorded by three Trimble R9 receivers equipped with choke ring antennas on the roofs of two buildings with different heights. Data collected on 19 October 2016 with a sampling interval of 0.5 s were used for the experiment. Figure 4 shows the observation environment of the three stations (Dataset 1, Dataset 2 and Dataset 3). G, C, R were the abbreviation of GPS, BDS and GLONASS. Stations A and B were located in a high multipath environment, while Station C was set in an open area. The baselines of A–B and A–C were 29.08 m and 69.79 m, respectively. Details of the dataset are summarized in Table 1. Figure 5 shows the locations of the three stations.

Another dataset (Dataset 4) was observed on 27 October 2016. This dataset used the same length data after eight earth rotation rates when compared with the dataset mentioned above. This dataset was collected at Station A, and the other detail parameters were the same.

**Figure 6:**
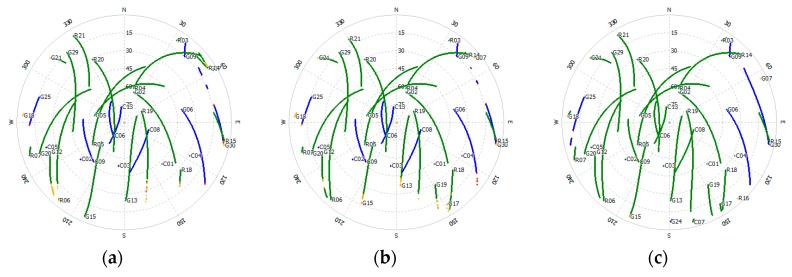
Sky plot of the three sites: (**a**) Site A, (**b**) Site B, (**c**) Site C.

**Figure 7:**
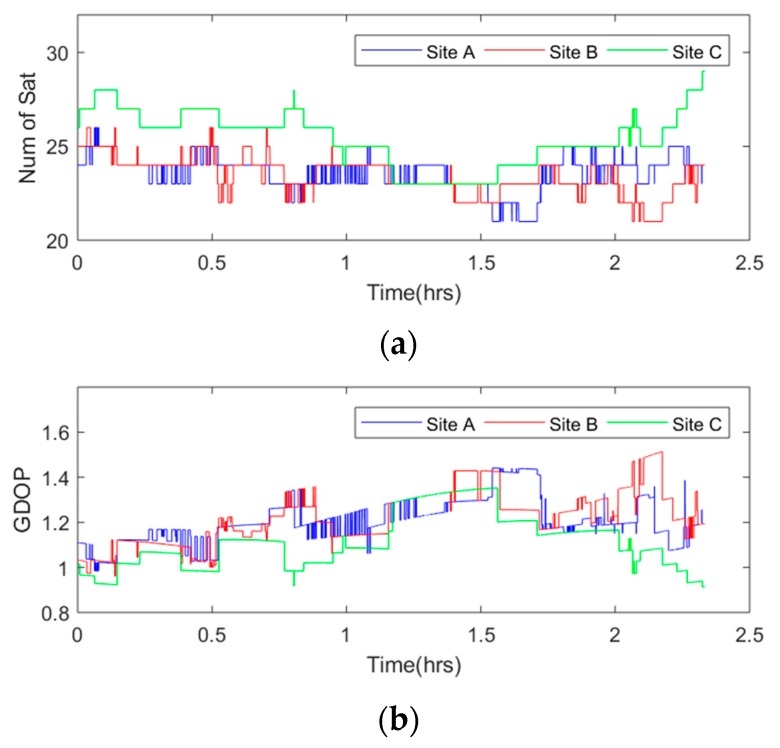
Number of visible satellites and GDOP values during the data collection. (**a**) Number of visible satellites, (**b**) GDOP values.

Figure 6 shows the sky plot of the three sites. From this figure, we could see that most satellites observed had the same trajectory while several satellites, such as G15 and R15, did not. Satellites observed stably and similarly were used in this experiment. In Figure 7, the number of visible satellites during data collection is shown in the top image, and the Geometric Dilution of Precision (GDOP) value during data collection is shown in the bottom panel. The average number of visible satellites at Site A was 24, with a maximum of 26 and a minimum of 21. The average number of visible satellites at Site B was 23, with the same maximum and minimum as those at Site A. The average number of visible satellites at Site C was 25, with a maximum of 29 and a minimum of 23. Since more visible satellites were observed at Site C, the Dilution of Precision (DOP) value was slightly lower than the values at Sites A and B, with a mean value of 1.11 to 1.20 and 1.22.

## 4. Results and Analysis

### 4.1. CMCD Spectral Analysis and Comparison

**Figure 8:**
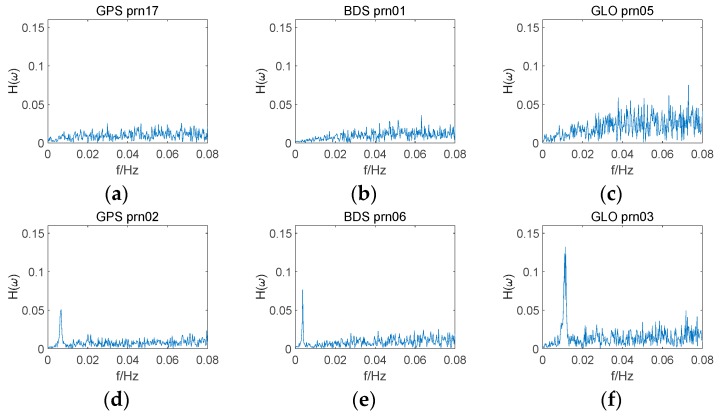
Comparison of the GNSS satellites’ frequency spectra, (**a**) G17, (**b**) C01, (**c**) R05, (**d**) G02, (**e**) C06, (**f**) R03.

Figure 8 shows the CMCD frequency spectrums for the GPS, GLONASS, and BDS satellites. It was noticed that large peaks appeared in the low frequency for G02, C06, and R03, while the CMCD frequency spectrums of G17, C01, and R05 were not seriously influenced by frequency peaks. Comparing the satellites with spectral peaks and the satellites without spectral peaks, the main spectral features appeared in the low-frequency spectrum. Compared with GPS and BDS, GLONASS satellites showed a significantly larger high-frequency noise. As for the satellites affected by the spectral peaks, the peak positions and peak heights were different among the different satellites, which means that different CMCD measurements suffered from different period error and the level of fluctuation may also be different.

**Figure 9:**
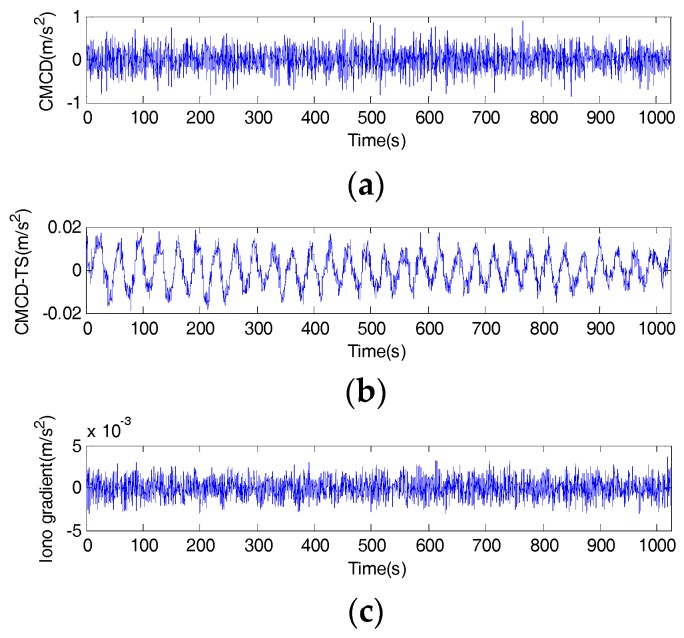
(**a**) Time series of the code-minus-carrier divergence (CMCD), (**b**) Time series of time-averaged CMCD, (**c**) Time series of ionospheric gradient.

For a better understanding of the features of this error, we plotted the time series of CMCD, time-averaged CMCD, and ionospheric gradient in Figure 9. Taking C06 as an example, the time series of CMCD performed as random noise. However, when we used the time-averaging method to eliminate the influence of high-frequency noise, the waveform fluctuated periodically. It is well known that the CMCD measurement is the first-order derivative of pseudorange measurements. Conversely, when the integration algorithm is used for white noise, the noise turns out to be amplified noise, and the periodic fluctuation turns into amplified periodic fluctuation. Due to the different noise characteristics with white noise, this kind of period error cannot be eliminated well by time-averaging smoothing, FIR, wavelet denoise, etc. in complex situations. To analyze whether this error was related to the ionospheric gradients, we used an ionospheric-free combination from adjacent epochs to obtain the ionospheric gradients. Results showed that the value of the ionospheric gradient reached ±0.002 m/s^2^. However, the range of the periodic waveform varied from −0.02 to 0.02 m/s^2^, which was much larger than that of the ionosphere gradient. Concerning the expression of Equation (7), we can conclude that the periodic fluctuations were most likely caused by multipath, rather than ionospheric gradients. 

**Figure 10:**
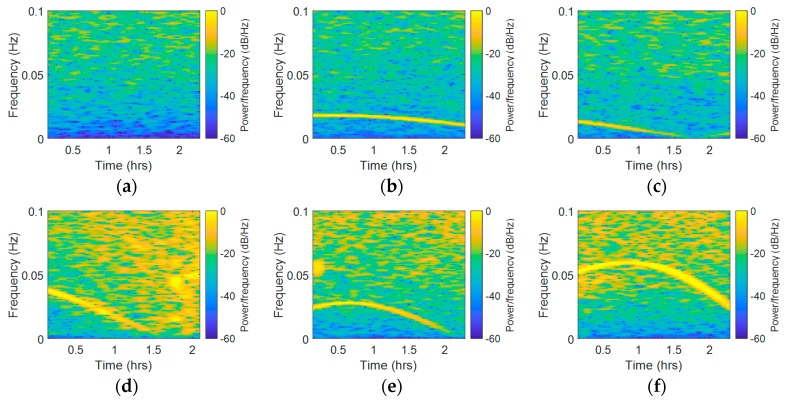

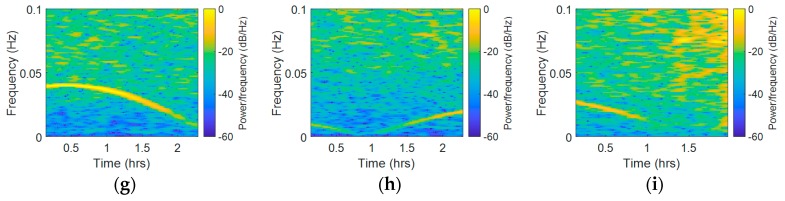
Short-time Fourier transformation (STFT) results of the nine satellites from BDS, GLONASS and GPS systems (Site A, (**a**) C01, (**b**) C06, (**c**) C08, (**d**) R04, (**e**) R05, (**f**) R20, (**g**) G02, (**h**) G05, and (**i**) G06).

Although fast Fourier transform (FFT) is appropriate for frequency domain analysis, it obtains only the frequency components of the overall signal, which means that this method cannot handle time-frequency analysis [39,40,41]. By dividing the signal into small sequential or overlapping data frames and applying FFT to each sequential, STFT is able to analyze the spectral variation over time. In this paper, STFT was used to obtain the time-frequency analysis of the CMCD features. The x-axis represents the time component, while the vertical axis represents the frequency spectrum component. Taking Site A as an example, the corresponding results are shown in Figure 10. Due to space limitations, herein we selected three satellites from each system. The STFT results of C01, C06, C08, R04, R05, R20, G02, G05, and G06 are presented in sequence. In STFT it changed from −50~−60 dB/Hz to −10~−20 dB/Hz in the vertical axis without the influence of frequency peaks, while the color changed from blue to yellow. If there was a frequency peak, we could clearly see a yellow line in the low frequency. Results showed that different satellites had different periods of fluctuation. The STFT results of C01 were not affected by this periodic waveform, and all the other Geostationary Earth Orbit (GEO) satellites had similar results. However, C06 and C08 were affected by this kind of error. Furthermore, we could see a clear yellow line ranging from 0~0.02 Hz. In most of the GLONASS satellites, we could also see this yellow line appearing in the STFT results, which ranged from 0~0.06 Hz in this experiment. For GPS satellites, it ranged from 0~0.04 Hz here. We also found that the waveform changed differently in different satellites. In G05, the yellow line in the figure rose and fell, and then rose again, which means that the waveforms fade away and then rise again quickly; this phenomenon also appeared in several BDS satellites such as C08. In some satellites such as C06, the yellow line changed slightly from 0.0125 Hz to 0.01 Hz, while in some satellites such as R08 and G02, the yellow line changed rapidly from 0.2 Hz to 0.6 Hz. The overall frequency spectrum was also different among the different systems. As it had more yellow areas, we concluded that the GLONASS satellites experienced a higher noise level than the other two systems. Moreover, the width of the yellow line in the GLONASS system was wider, indicating that the peaks in GLONASS were wider than those in GPS and BDS systems. The difference of the overall frequency spectrums may be related to the fact that the Frequency Division Multiple Access (FDMA) strategy is used by GLONASS, while the other systems adopt the Code Division Multiple Access (CDMA) strategy. In R04 and G06, the STFT frequency spectrums in the start time and end time were different, which means that the noise frequency spectrum was also uncertain. Overall, it can be seen that the peaks width, frequency, and strength as well as the overall frequency spectrum strength varied with satellites and time. This time-varying character might be referred to some other systematic errors, which indicates great difficulty in eliminating this kind of error adaptively and effectively.

**Figure 11:**
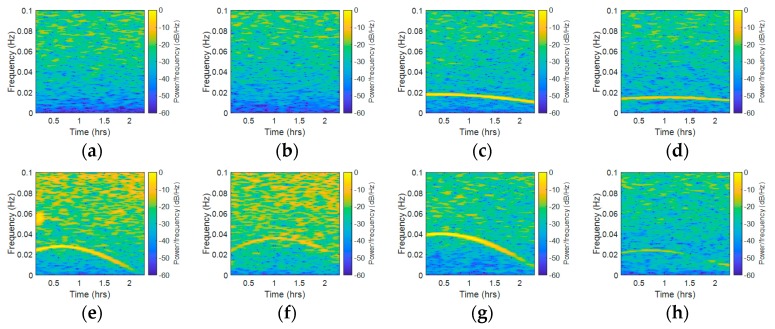
STFT results of Site A and Site B (baseline 29.08 m), (**a**) Site A, C01, (**b**) Site B, C01, (**c**) Site A, C06, (**d**) Site B, C06, (**e**) Site A, R05, (**f**) Site B, R05, (**g**) Site A, G02, (**h**) Site B, G02.

**Figure 12:**
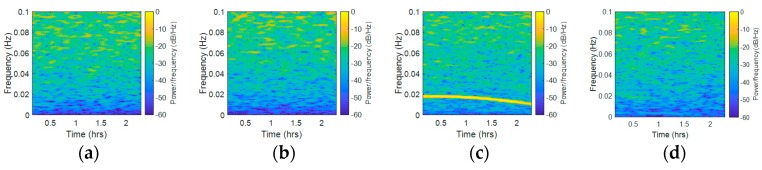

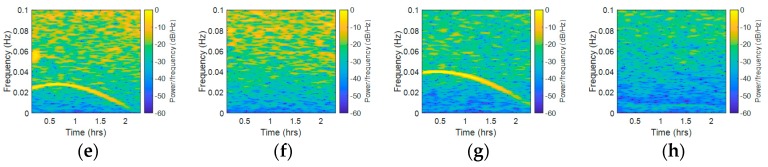
STFT results of Site A and Site C (baseline 69.79 m), (**a**) Site A, C01, (**b**) Site C, C01, (**c**) Site A, C06, (**d**) Site C, C06, (**e**) Site A, R05, (**f**) Site C, R05, (**g**) Site A, G02, (**h**) Site C, G02.

To further analyze whether this kind of error was highly correlated with the observation environment, we compared the STFT results from different stations. Figure 11 shows the STFT results of Sites A and B during the same observation period, while Figure 12 shows the STFT results of Sites A and C during the same observation period. Figure 11a,c,e,g present the STFT results of C01, C06, R05, and G02 measured at Site A, while Figure 11b,d,f,h present the STFT results for the same satellites measured at Site B. Figure 12b,d,f,h present the STFT results for the same satellites measured at Site C. According to Figure 11, the results showed that the BDS GEO satellite was always unaffected by this periodic error. In the comparison to other satellites, the results showed that the peaks width and frequency varied with different receivers. From Figure 11c,d, we can conclude that the changes of waveforms had a slight difference to each other. In Figure 11e,f, the difference became more significant. In Figure 11g,h, the peak width and strength had an obvious difference. According to Figure 12, one may notice that the results from Site A were subject to periodic error, while the results from Site C were not affected by such periodic error. These results also verified that multipath is obviously affected by the environment. In all of the comparison groups, the results showed that the same satellite observed by different receivers had similar overall frequency spectrum strengths, but the overall frequency spectrum strengths were different in different systems.

**Figure 13:**
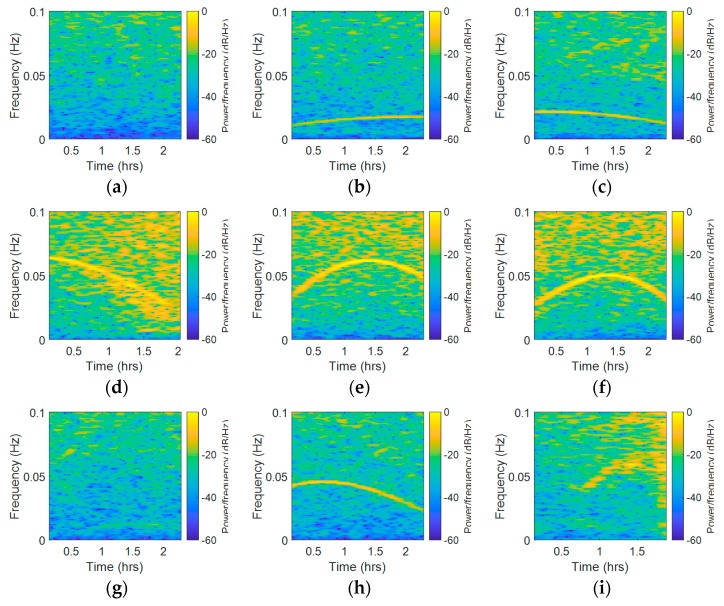
STFT results of Dataset 4, observed on 27 October 2016, Site A, (**a**) C01, (**b**) C06, (**c**) C08, (**d**) R04, (**e**) R05, (**f**) R20, (**g**) G02, (**h**) G05, and (**i**) G06.

In order to avoid suspicions another dataset observed on 27 October 2016 was analyzed. The results are shown in Figure 13. In Figure 13, the STFT results of C01, C06, C08, R04, R05, R20, G02, G05, and G06 are presented in sequence. There were eight earth rotation rates (23 h, 56′4″) between the observation time on 19 and 27 October 2016. From Figure 13, our conclusions that the BDS GEO satellite was always unaffected by this periodic error and that GLONASS satellites experienced a higher noise level than the other two systems still hold true. Comparing Figure 13 with Figure 10, we found that the variation trend in most satellites had different behaviors. Based on these behaviors, we concluded that the period fluctuations had low correlation with the earth rotation rate.

**Figure 14:**
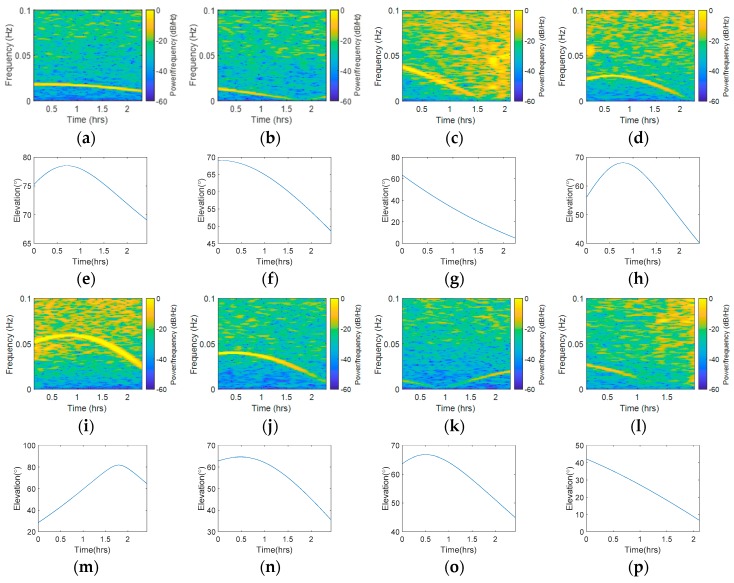
STFT and elevation results of Site A, (**a**) C06, STFT, (**b**) C08, STFT, (**c**) R04, STFT, (**d**) R05, STFT, (**e**) C06, Elevation, (**f**) C08, Elevation, (**g**) R04, Elevation, (**h**) R05, Elevation, (**i**) R20, STFT, (**j**) G02, STFT, (**k**) G05, STFT, (**l**) G06, STFT, (**m**) R20, Elevation, (**n**) G02, Elevation, (**o**) G05, Elevation, (**p**) G06, Elevation.

To find out whether there was a link between the abnormal frequency spectrum and elevation angle, we compared the variation tendency of the yellow line in the STFT results and elevation in Figure 14. The same satellites as those shown in Figure 10 were chosen, except C01. Figure 14a–d and 14i–l present the STFT results of C06, C08, R04, R05, R20, G02, G05, and G06 measured at Site A, while Figure 14e–h and 14m–p present the elevation figures at the same time of the corresponding satellites. The abnormal frequency spectra of C01, R04, R05, G02, and G06 exhibited a similar tendency with elevation. Nevertheless, the tendency rose and fell, then rose again in C08 and G05, which presented a low similarity to the elevation trends. In R20, the time of the highest frequency peak that appeared in STFT was different with the time of the highest frequency peak that appeared in the elevation figure; moreover, the correlation of the STFT variation tendency was also low. In a word, some satellites’ spectrum peaks variation tendency in the STFT results had high similarity to elevation, while some satellites’ results did not. A possible explanation of these results is that the elevation angle is one factor that influences the results, but not the only factor. In addition, there was an association between the range of elevation angles and the range of abnormal peak frequency. In C06 and C08, the range of elevation angles is relatively narrow (9.5° and 20.6°), while the abnormal peak frequency is stable. In R04, R20, and G02, the range of elevation angles is wide (58.4°, 53.4°, and 29.2°), so the frequency variation range of abnormal is correspondingly wide.

### 4.2. Results and Analysis of the Proposed Method

In this section, STFT was used to analyze the features of the measured data and the results are shown according to the different systems.

**Figure 15:**
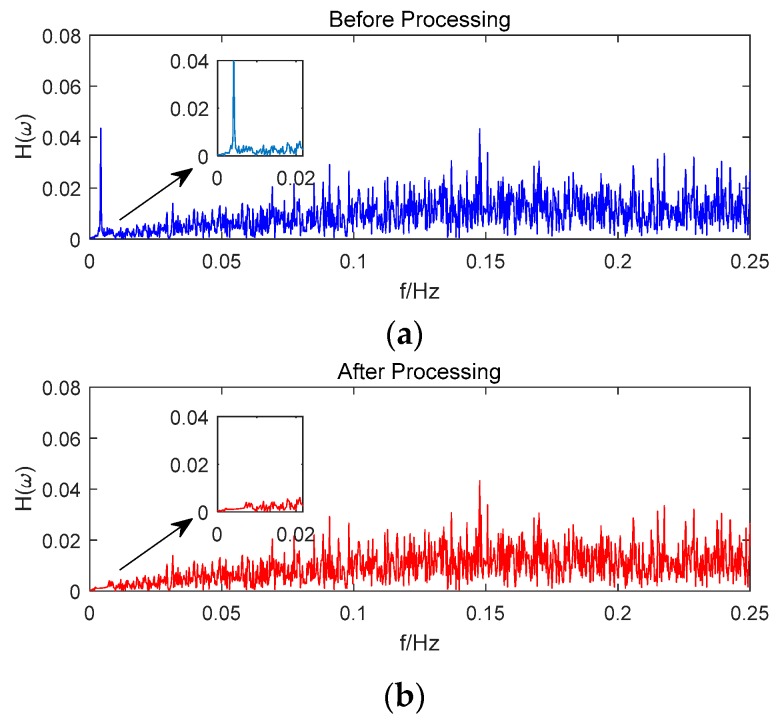
(**a**) Frequency spectrum before smoothing with the spectrum peaks extracting method and CMCD correction model, C06, (**b**) Frequency spectrum after smoothing with the spectrum peaks extracting method and CMCD correction model, C06.

**Figure 16:**
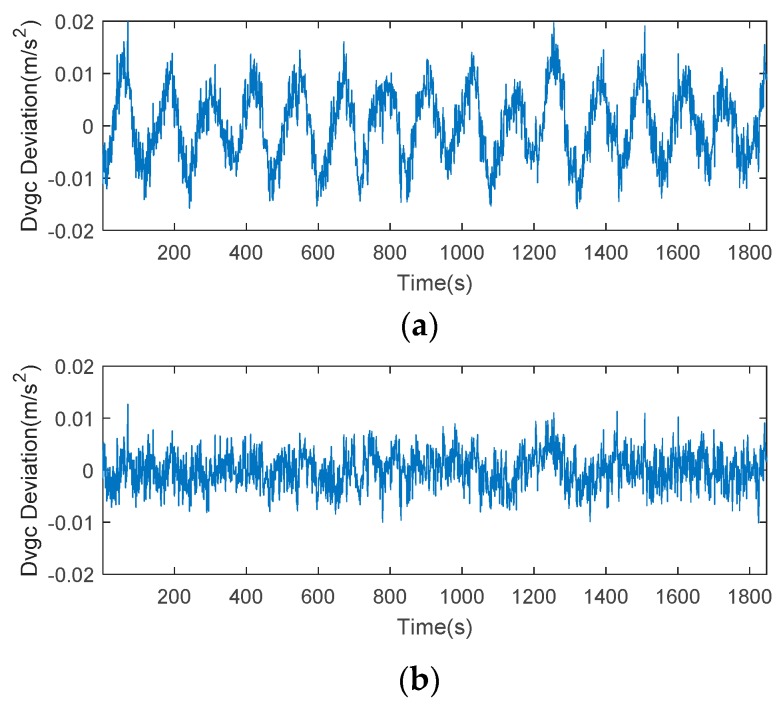
(**a**) CMCD (Time averaging, 100 epochs) in the time domain comparison before the adaptive frequency domain filter, C06, (**b**) CMCD (Time averaging, 100 epochs) in the time domain comparison after the adaptive frequency domain filter, C06.

**Figure 17:**
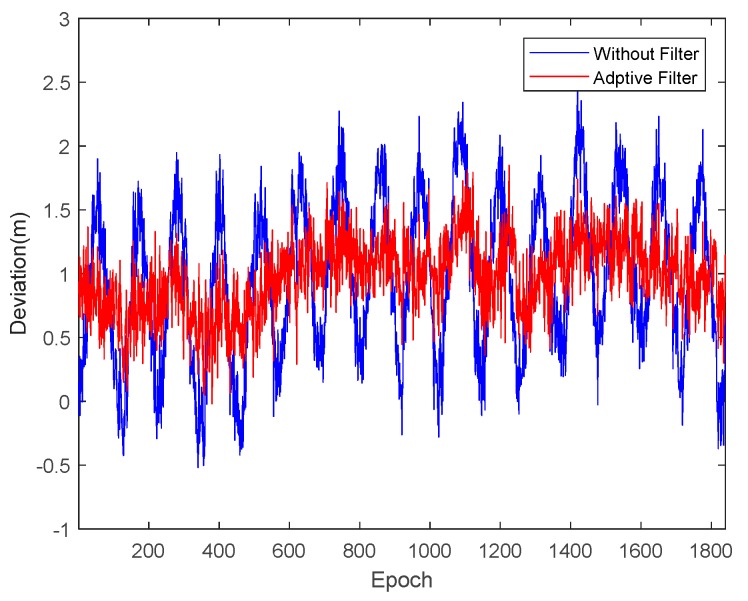
Pseudorange residuals comparison before and after frequency spectrum smoothing (C06, 1800 s).

**Figure 18:**
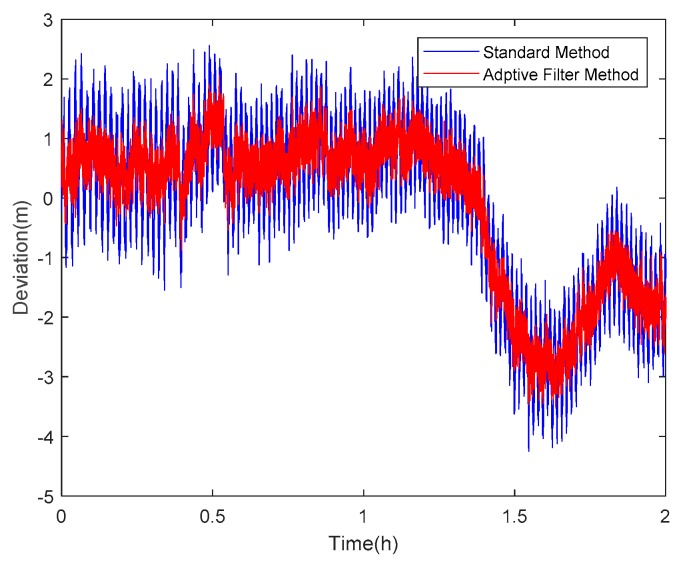
Pseudorange residuals comparison before and after frequency spectrum smoothing (C06, 2 h).

Taking satellite C06 as an example of the BDS system, Figure 15 shows the frequency spectrum before and after smoothing with the spectrum peaks extracting method and CMCD correction model. Figure 16 shows the CMCD results before and after the implementation of the adaptive frequency domain filter. Figure 17 shows the pseudorange residuals before and after smoothing. Figure 18 presents the 2 h result before and after frequency spectrum smoothing, where the results showed that the proposed method could completely eliminate the influence of frequency peaks. Hence, the periodic error could be clearly eliminated in the time series, meaning that the error could be efficiently reduced. As is shown in Figure 9, the data influenced by the high noise frequency spectrum performed as random noise. In order to eliminate the influence of high frequency noise and better present the influence of this frequency domain filter method, the time-averaging method was used in the CMCD series before and after processing, just for display. In Figure 15, the difference in the low frequency spectrum before and after smoothing was highlighted in a sub-graph, indicating that the abnormal frequency peak was eliminated after smoothing. As seen from Figure 16, periodic waveforms could be observed before filtering using the time-averaging method (100 epochs), while the periodic waveforms were significantly removed after filtering. As is widely known, the CMCD series is the first-order difference of the combination of the pseudorange and carrier phase, so the low frequency period fluctuation in CMCD would be amplified in the pseudorange after integration. In Figure 17 and Figure 18, the results show that the former period fluctuation in the pseudorange reached up to 1.25 m. After the filter process, the fluctuation decreased to about 0.5 m. The majority of the period influence was eliminated, and the processed pseudorange noise performed like white noise. 

**Figure 19:**
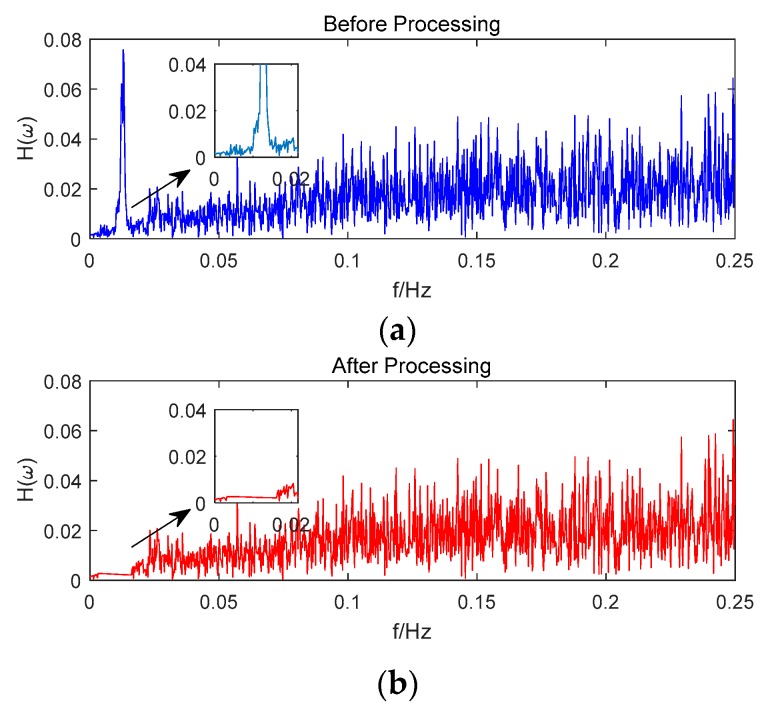
(**a**) Frequency spectrum before smoothing with the spectrum peaks extracting method and CMCD correction model, R20, (**b**) Frequency spectrum after smoothing with the spectrum peaks extracting method and CMCD correction model, R20.

**Figure 20:**
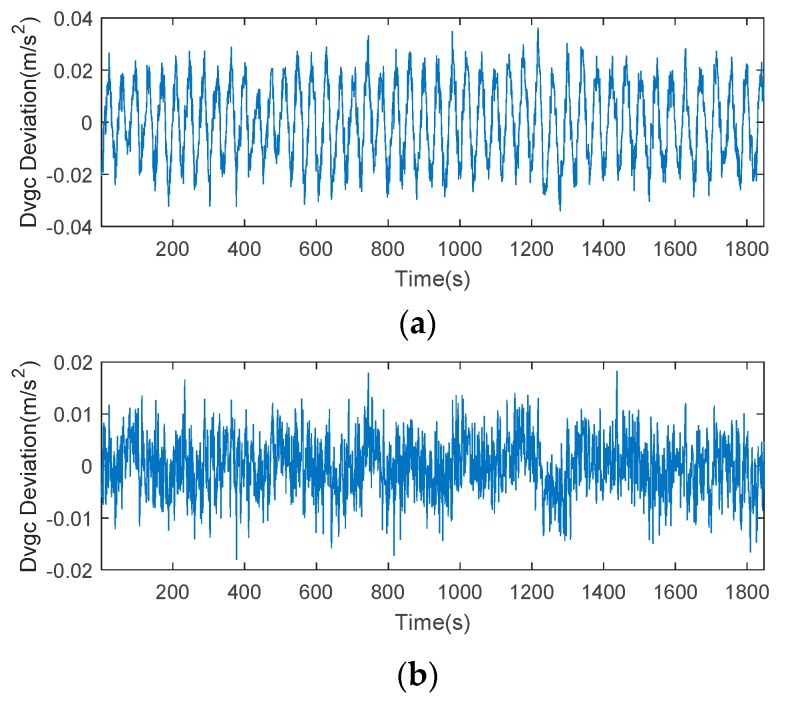
(**a**) CMCD (Time averaging, 100 epochs) in the time domain comparison before the adaptive frequency domain filter, R20, (**b**) CMCD (Time averaging, 100 epochs) in the time domain comparison after the adaptive frequency domain filter, R20.

**Figure 21:**
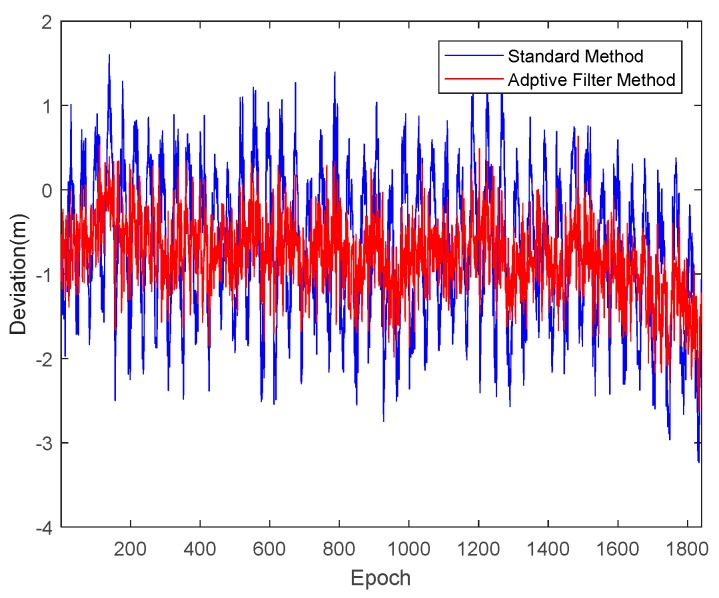
Pseudorange residuals comparison before and after frequency spectrum smoothing (R20, 1800 s).

**Figure 22:**
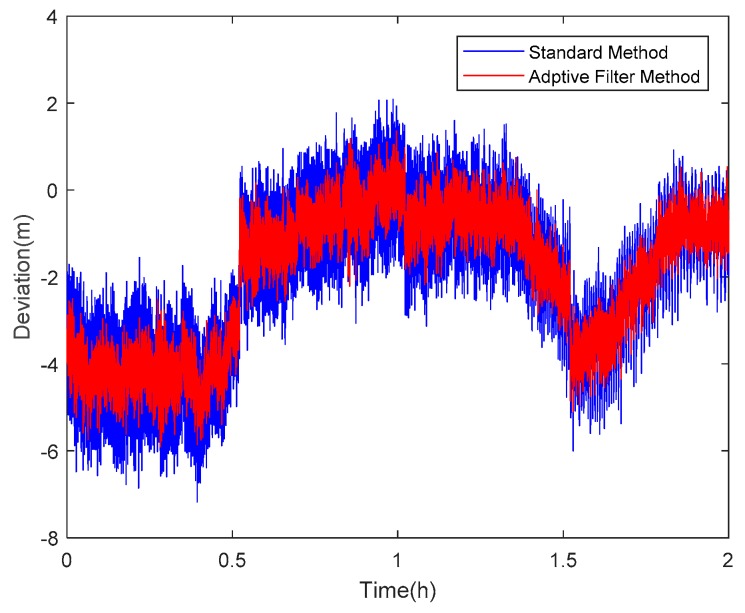
Pseudorange residuals comparison before and after frequency spectrum smoothing (R20, 2 h).

Using satellite R20 as an example of the GLONASS system, Figure 19 shows the frequency spectrum before and after smoothing with the spectrum peaks extracting method and CMCD correction model. Figure 20 shows the CMCD results before and after the implementation of the adaptive frequency domain filter. Figure 21 shows the pseudorange residuals before and after smoothing. In Figure 22, we present the 2 h result before and after frequency spectrum smoothing. The difference in the low frequency spectrum before and after smoothing was highlighted in a sub-graph of Figure 19, indicating that the abnormal frequency peak was eliminated after smoothing. With the proposed adaptive smoothing filter, the frequency peaks disappeared and the effect of the periodic fluctuations was greatly reduced. As seen from Figure 20, periodic waveforms could be observed before filtering using a time-averaging method (100 epochs), while the periodic waveforms were significantly removed after filtering. Similarly, the pseudorange errors were significantly decreased from 2 m to 1 m in Figure 20 and Figure 21.

**Figure 23:**
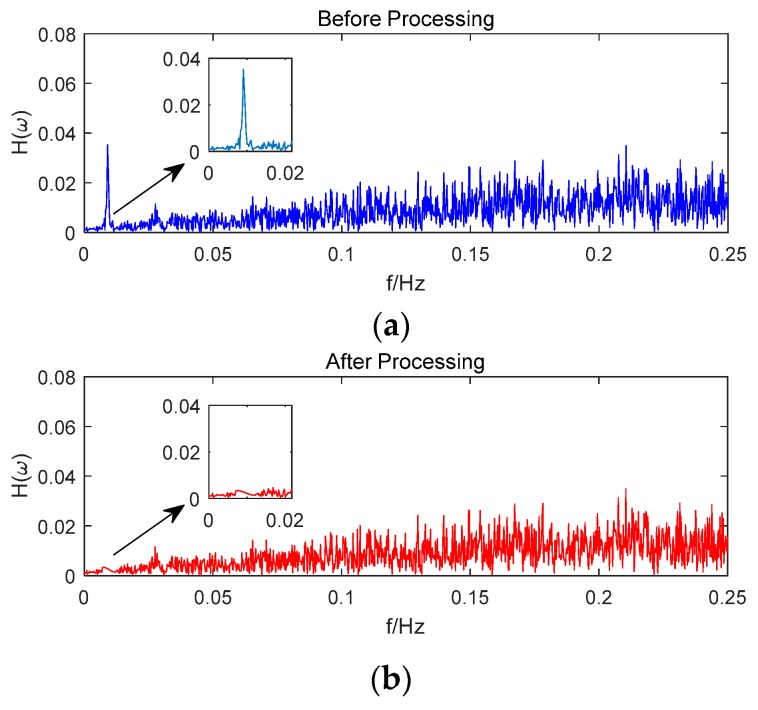
(**a**) Frequency spectrum before smoothing with the spectrum peaks extracting method and CMCD correction model, G06, (**b**) Frequency spectrum after smoothing with the spectrum peaks extracting method and CMCD correction model, G06.

**Figure 24:**
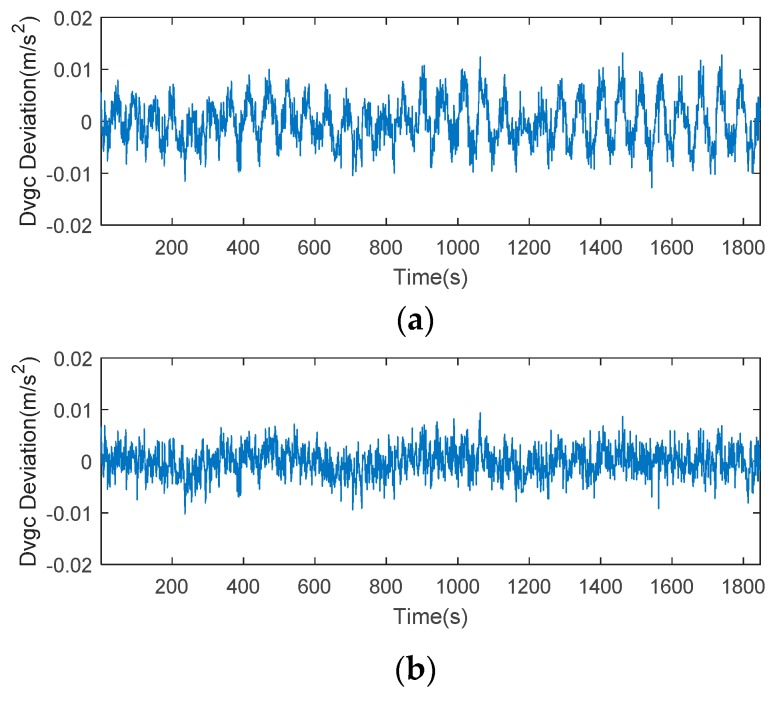
(**a**) CMCD (Time averaging, 100 epochs) in the time domain comparison before the adaptive frequency domain filter, G06, (**b**) CMCD (Time averaging, 100 epochs) in the time domain comparison after the adaptive frequency domain filter, G06.

**Figure 25:**
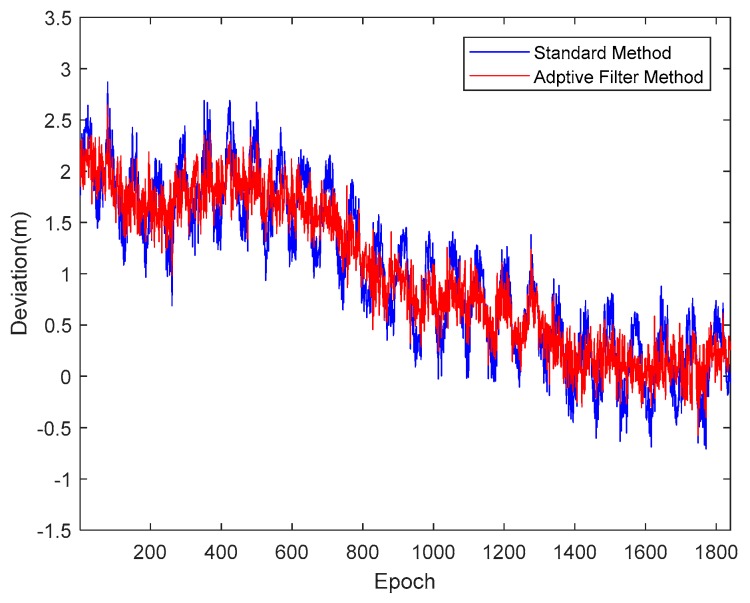
Pseudorange residuals comparison before and after frequency spectrum smoothing (G06, 1800 s).

**Figure 26:**
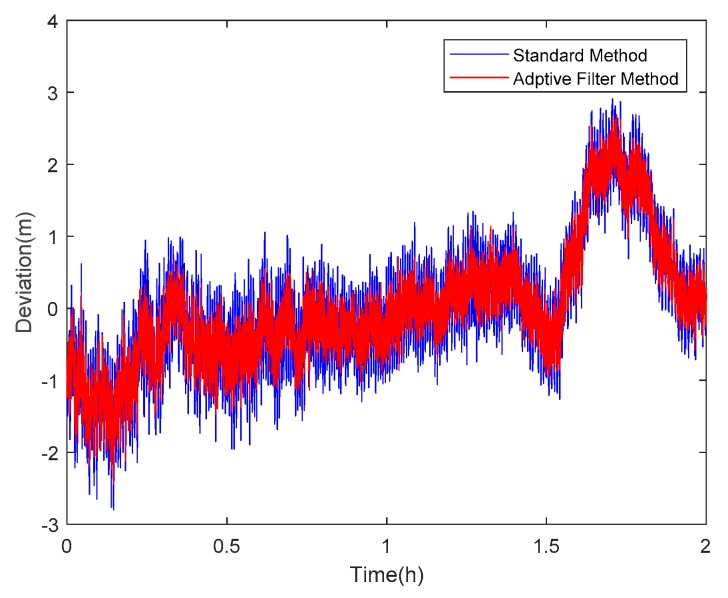
Pseudorange residuals comparison before and after frequency spectrum smoothing (G06, 2 h).

Likewise, taking satellite G06 as an example of the GPS system, Figure 23 shows the frequency spectrum before and after smoothing with the spectrum peaks extracting method and CMCD correction model. Figure 24 shows the CMCD results before and after the implementation of the adaptive frequency domain filter. Figure 25 shows the pseudorange residuals before and after smoothing. In Figure 26, the 2 h result before and after frequency spectrum smoothing is given. In Figure 23, the difference in the low frequency spectrum before and after smoothing was highlighted in a sub-graph, indicating that the abnormal frequency peak was eliminated after smoothing. With the proposed adaptive smoothing filter, the frequency peaks disappeared and the effect of periodic fluctuations was greatly reduced. As seen from Figure 24, periodic waveforms could be observed before filtering using a time-averaging method (100 epochs), while the periodic waveforms were significantly removed after filtering. Similarly, the pseudorange errors were significantly decreased from 2 m to 1 m in Figure 25 and Figure 26. The results of the GPS satellites were similar to those of BDS and GLONASS, indicating that the influence of periodic errors was efficiently mitigated with the proposed method.

It is worth mentioning that the pseudorange residuals suffer from different systematic errors for different satellites. It is rather difficult to quantify the smoothness obtained within the influence of fixed deviations. In this paper, higher-order fitting was used to extract the basic signal trend. To verify the improvement of the proposed method, the standard deviations of the pseudorange residuals were used for comparison. Since the parameters of different kinds of methods influence the filter results, for example, the time-averaging constant, FIR window type and cut-off frequency, wavelet basis, etc., the original pseudorange residuals were used as a comparison for this kind of frequency-domain filter. For a fair comparison, both high-frequency noises were kept in this experiment. Due to space limitations, the results of some typical satellites are shown in Table 2. For the BDS GEO satellites (C01~C05), the standard deviations were approximately 0.2~0.3 m, together with an improvement of about 0.1~0.2% for the proposed method, indicating that the GEO satellites were almost free from the periodic effects. For the BDS inclined geosynchronous satellite orbit (IGSO) satellites (C06, C08, C09), the standard deviations without filtering reached 0.6~0.7 m, while the standard deviations with the proposed method were 0.2~0.3 m, except for C08. The average improvement reached 50~60%, which means that the periodic multipath effect could be greatly reduced by the proposed method. Results of the BDS MEO (C13) were similar to those of the IGSO satellites. For the GLONASS satellites, the noise level of pseudorange was significantly higher than those of GPS and BDS. The standard deviations with a traditional method ranged from 0.6 to 1.0 m, while the standard deviations decreased to 0.4~0.8 m with an improvement of 20~50%. For GPS satellites, most of the standard deviations decreased from 0.3~0.5 m to 0.2~0.3 m once the proposed method was applied for multipath mitigation.

## 5. Conclusions

The multipath effect is generated when a signal arrives at the antenna through multiple paths instead of one direct path. Despite all the effort put into mitigating multipath errors, it remains the dominant error source that cannot be ignored for GNSS precise positioning and other GNSS applications. In this paper, we first analyzed the frequency spectrums of the code-minus-carrier divergence (CMCD) for GPS, GLONASS, and BDS satellites. It was found that frequency peaks and periodic fluctuations appeared in the GPS, GLONASS, and BDS MEO/IGSO satellites. Compared with GPS and BDS, GLONASS satellites showed a significantly larger high-frequency noise. As for the satellites affected by spectral peaks, the peak positions and peak heights were different among different satellites, which means that different CMCD measurements suffer from different period errors and the level of fluctuation may also be different. To eliminate the frequency peaks and period fluctuations caused by the multipath effect, a new method for GNSS multipath mitigation with adaptive frequency domain filters was proposed. Results showed that the proposed method could effectively eliminate the influence of frequency peaks and the periodic multipath effects were greatly reduced.

## Figures and Tables

**Table 1:** Details of the datasets.

	Dataset 1	Dataset 2	Dataset 3	Dataset 4
**Station**	**A**	**B**	**C**	**A**
Receiver	Trimble R9	Trimble R9	Trimble R9	Trimble R9
Antenna	Choke ring	Choke ring	Choke ring	Choke ring
Sampling Interval/s	0.5	0.5	0.5	0.5
Cut-off angle/°	5	5	5	5
Systems	G/C/R	G/C/R	G/C/R	G/C/R
Baseline/m	0	29.08	69.79	0
Observation date	19 October	19 October	19 October	27 October
Time/hour	2.5	2.5	2.5	2.5
Environment	Partial occlusion	Partial occlusion	Open environment	Partial occlusion

**Table 2:** Standard deviation of pseudorange residuals.

System	Prn	Without Filter	Proposed Method (m)	Improvement Percentage (%)
BDS	01	0.2510	0.2507	0.1
BDS	02	0.3272	0.3264	0.2
BDS	03	0.2765	0.2763	0.08
BDS	04	0.2876	0.2873	0.1
BDS	05	0.3685	0.3684	0.05
BDS	06	0.6714	0.2562	61.84
BDS	08	0.7636	0.6060	20.64
BDS	09	0.6651	0.3182	52.16
BDS	13	0.5050	0.2817	44.22
GLO	04	1.0315	0.8321	19.34
GLO	05	0.6747	0.5780	14.34
GLO	18	0.6074	0.4613	24.05
GLO	19	0.8938	0.4404	50.73
GLO	20	0.9260	0.7013	20.23
GPS	02	0.4392	0.2713	38.22
GPS	05	0.3474	0.2777	20.07
GPS	06	0.4215	0.3102	26.41
GPS	13	0.3993	0.2359	40.90
GPS	25	0.5735	0.4643	19.05
GPS	29	0.4333	0.2844	34.37
